# Exogenous Melatonin Attenuates Post-Harvest Decay by Increasing Antioxidant Activity in Wax Apple (*Syzygium samarangense*)

**DOI:** 10.3389/fpls.2020.569779

**Published:** 2020-09-11

**Authors:** Yao Chen, Yanjie Zhang, Ghazala Nawaz, Chenxu Zhao, Yuxia Li, Tingting Dong, Mingku Zhu, Ximeng Du, Lei Zhang, Zongyun Li, Tao Xu

**Affiliations:** ^1^ Jiangsu Key Laboratory of Phylogenomics and Comparative Genomics, School of Life Sciences, Jiangsu Normal University, Xuzhou, China; ^2^ School of Agricultural Sciences, Zhengzhou University, Zhengzhou, China; ^3^ Department of Botanical and Environmental Sciences, Kohat University of Science and Technology, Kohat, Pakistan

**Keywords:** wax apple, exogenous melatonin, post-harvest, preservation, decay, antioxidation

## Abstract

Wax apple is one of the most popular tropical fruit but undergoes serious post-harvest decay during storage, transportation and marketing. Melatonin (MT) plays important roles in plant growth, development and stress responses. However, its function in post-harvest preservation of fruit remains largely unknown. In the present study, the physiological function and molecular mechanism of exogenous MT for post-harvest preservation were evaluated in wax apple fruit. Results showed that MT treatment remarkably reduced decay incidence and the accumulation of excess reactive oxygen species (ROS) but increased the activity of antioxidant enzymes, suggesting that exogenous MT alleviates the post-harvest decay of wax apple by regulating the balance between ROS production and antioxidant system. Meanwhile, the gene expression was analyzed by transcriptome confirmed by quantitative PCR. This study provides insights into the regulatory mechanism and proper application strategies for post-harvest preservation of wax apple and other fruits though melatonin manipulation.

## Introduction

Wax apple (*Syzygium samarangense*) is an important tropical cash crop in Southeast Asian countries, including Thailand, Malaysia and Taiwan (Shü et al., 2008). The wax apple is an immunostimulant and antibacterial drug in traditional medicine. The extract from leaves of wax apple contains many compounds including condensed tannins, flavonoids, ellagitannins and phenolic acids, and exhibits a noticeable antioxidant activity (Sobeh et al., 2018). For instance, the compound myricitrin from wax apple leaves can mitigate negative effects from oxidative stress by modulating the mitogen-activated protein kinase (MAPK) signaling pathways, and another compound 3,5-di-O-methyl gossypetin exerts its antioxidant activity through the MAPK signaling pathway and the nuclear transcription factor-2 (Nrf-2) pathway (Sobeh et al., 2019). In addition, the essential oil compositions in the wax apple leaves have been investigated by using GLC/MS and GLC/FID (Sobeh et al., 2016). Especially, wax apple fruit has become increasingly popular in Western countries because of its unique flavor. However, as a typical nonclimacteric fruit, wax apple cannot be further ripened following harvest; hence, wax apples should only be harvested when fully ripe (Moneruzzaman et al., 2011). Additionally, wax apple has a thin pericarp and abundant water content; thus, it might suffer physical damage and cracks during transportation and become brown and perishable (Supapvanich et al., 2012). Wax apple can only be kept in storage for a week at ambient temperature (Supapvanich et al., 2018).

Several methods for post-harvest preservation of wax apple have been explored and applied. Low-temperature storage is the most common method, in which the antioxidant and ascorbic acid (AsA) capacity of wax apple is allowed to increase accordingly with the decrease in storage temperature (Supapvanich et al., 2011). However, chilling damage may occur if the temperature is too low. Applying fresh *Aloe vera* gel or konjac glucomannan coating incorporated with pineapple core extract to fresh-cut wax apples can delay the incidence of browning and maintain the colour of the fruit during storage (Supapvanich et al., 2012; Supapvanich et al., 2016). However, more approaches for efficient post-harvest preservation of wax apples should be explored and evaluated.

Melatonin (MT), known as N-acetyl-5-methoxytryptamine, is an indole compound that widely exists in animals and plants (Arnao and Hernández-Ruiz, 2015). MT plays an important role in improving sleep and has anti-aging and anti-inflammation properties (Dubbels et al., 1995). In higher plants, MT participates in various physiological functions, including light signal regulation, growth regulation, and biotic and abiotic stress adaptations (Zhang et al., 2013; Liang et al., 2015). MT can act as an effective free radical scavenger and antioxidant to maintain the lipid membrane structure and protein content of tissues, thereby avoiding free radical damage (Reiter et al., 1998). Recently, the role of MT in post-harvest processes has been elucidated in peach (Cao et al., 2018; Gao et al., 2018), banana (Hu et al., 2017), strawberry (Aghdam and Fard, 2017), cucumber (Xin et al., 2017), cassava (Ma et al., 2016), and tomato (Sun et al., 2016). Thus, MT can be a potential target for improving the post-harvest preservation of fruits and vegetables (Xu et al., 2019). However, the function of exogenous MT in post-harvest preservation of wax apple remains unknown.

In this study, we investigated the alleviative effects of MT on the deterioration of post-harvest wax apple and demonstrated its mechanism of action. Analyses of decay incidence, weight loss, and oxidation-related indicators and antioxidant enzyme contents, such as jasmonic acid (JA) and salicylic acid (SA), were conducted. Gene expression was investigated *via* transcriptome sequencing and quantitative polymerase chain reaction (PCR). Gene Ontology (GO) enrichment was also analysed. Our study will elucidate the post-harvest preservation of wax apple and help understand the role of MT in post-harvest fruit preservation.

## Materials and Methods

### Plant Materials and Treatments

Wax apples (*Syzygium samarangense* cv. ‘Heijingang’) were harvested from an orchard and then transported to the laboratory. The harvested fruit without defects and injuries, in commercial maturity and with average weight of 76 ± 1.8 g were selected for subsequent experiments. The selected fruits were soaked in 1% sodium hypochlorite for 5 min and rinsed with running water. The fruits were randomly divided into different treatment groups with 51 fruits each. The optimum MT concentration (800 µM) was determined after preliminary experiments with MT concentrations of 0, 100, 200, 400, and 800 µM. The MT treatment groups were immersed in deionized water (diH_2_O) with MT at 25°C for 2 h. The wax apples were exposed to low light conditions to prevent MT degradation (Gao et al., 2018). After melatonin or diH_2_O (as control) treatment, the solution on the surface of wax apple was removed, and then the wax apple were air-dried approximately 10 min and stored for 7 days at 25 ± 1°C and 65–70% relative humidity. Morphological changes, decay incidence, and weight loss rate were recorded every day during storage. At the same intervals, the pericarp of wax apples was excised, and the pulp was processed into pieces, frozen by liquid nitrogen, and stored at –80°C for subsequent experiments.

### Decay Incidence and Weight Loss Rate

Visible decay, tissue damage and bacterial/fungal growth were considered decay. Decay incidence was expressed as the percentage of the visible spoilage of fruit relative to the total number of fruit in each treatment group. The treated fruits were weighed and recorded before and after storage, and weight loss rate was expressed as the percentage of weight loss compared with initial weight. All experiments were performed with three independent biological replicates.

### Determination of Reactive Oxygen Species and Malondialdehyde Contents

Superoxide radical (O_2_^−^) production rate was detected following a previously described method (Wang and Luo, 1990) with slight modifications. Briefly, 2 g of wax apple pulp was homogenized in 2 mL of 50 mM sodium phosphate buffer (pH 7.8) containing 1 mM ethylene diamine tetraacetic acid (EDTA), 0.3% Triton X-100, and 2% polyvinylpyrrolidone (PVP) and then centrifuged at 12,000 × g at 4°C for 20 min. The supernatant was collected and used to determine the rate of O_2_^−^ production by measuring the absorbance at 530 nm. O_2_^−^ production rate was based on hydroxylamine oxidation and expressed as mol kg^−1^ prot on fresh weight (FW) basis. Hydrogen peroxide (H_2_O_2_) content was determined following a previously described protocol (Patterson et al., 1984). Briefly, 0.1 g of pulp sample was thoroughly homogenized in 0.9 mL of physiological saline and then centrifuged at 12,000 × g at 4°C for 20 min. The supernatant was collected and used for colorimetric determination at 412 nm. H_2_O_2_ content was determined according to the yellow precipitate generated by reaction with titanium tetrachloride and expressed as mmol kg^-1^ prot on FW basis. Malondialdehyde (MDA) content was determined *via* thiobarbituric acid method (Dhindsa et al., 1981). Briefly, 1 g of pulp sample was mixed with 5 mL of 5% (w/v) trichloroacetic acid solution and centrifuged at 10,000 × g at 4°C for 20 min. The mixture was heated for 20 min above 95°C, cooled rapidly and centrifuged at 5000 × g for 20 min. The corresponding absorbance value was measured at 532 nm, and non-specific absorbance values at 450 and 600 nm were subtracted. MDA content was expressed as μmol kg^-1^ on FW basis. All experiments were performed with three independent biological replicates.

### Determination of Enzyme Activity

Pulp samples (5 g) were homogehomogenized with 5 mL of various relevant buffers, which were precooled at 4°C. Liquid oxygen (LOX) were determined with 0.1 mol L^-1^ sodium phosphate buffer (pH 6.8) containing 1 mL of Triton X-100 and 5% PVP. Superoxide dismutase (SOD) was determined with 0.1 mol L^-1^ sodium phosphate buffer (pH 7.8) containing 5 mM DL-dithiothreitol (DTT) and 5% PVP. Catalase (CAT) was determined with 50 mM sodium phosphate buffer (pH 7.5) containing 5 mM DTT and 5% PVP. Ascorbate peroxidase (APX) was determined with potassium phosphate buffer (pH 7.5) containing 0.1 mM EDTA, 1 mM AsA, and 2% PVP. Glutathione reductase (GR) was determined with 0.1 mol L^-1^ sodium phosphate buffer (pH 7.5) containing 0.1 mM EDTA. The supernatants were collected for enzyme activity determinations.

CAT activity was measured by H_2_O_2_ consumption at the absorbance of 240 nm (Dhindsa et al., 1981). APX activity was determined by oxidizing AsA with H_2_O_2_ and estimating the decrease in absorbance at 290 nm (Nakano and Asada, 1989). GR activity was determined by oxidation–reduction reaction of nicotinamide adenine dinucleotide phosphate at the absorbance of 340 nm (Palma et al., 2006). One unit (U) of CAT, APX and GR activity represents the amount of enzyme required to reduce the reaction system by 0.01 per minute. LOX activity was determined by catalysing the formation of conjugated double bonds by sodium linoleate and estimating the increase in absorbance at 234 nm (Surrey, 1964). One unit (U) of LOX activity represents the amount of enzyme required to increase the reaction system by 0.01 per minute. SOD activity was determined by inhibiting the reduction of nitroblue tetrazolium (NBT) under light (Zhang et al., 2013). One unit (U) of SOD activity indicates the amount of enzyme required to inhibit the NBT photochemical reduction by 50% per minute at the absorbance of 560 nm. All enzyme activities were indicated as U g^−1^ based on FW. All experiments were performed with three independent biological replicates.

### Determination of Jasmonic Acid and Salicylic Acid Contents

Pulp sample (1 g) was homogenized with an appropriate amount of sodium phosphate buffer (pH 7.4) and then centrifuged at 5000 × g at 4°C for 20 min, and the supernatant was obtained for subsequent experiments. JA and SA contents were measured using enzyme-linked immunosorbent assay kits (Enzyme-linked Biotech, Shanghai, China) according to the manufacturer’s instructions. JA and SA contents were expressed as nmol L^-1^ and mg L^-1^ on FW basis, respectively. All experiments were performed with three independent biological replicates.

### *De Novo A*ssembly, Unigene Annotation, and Functional Classification

Total RNA was extracted from the 5th day samples (including control and treatment groups with three independent biological replicates) with Trizol reagent (Invitrogen, CA, USA). Six libraries for mRNA sequencing were constructed for Illumina HiSeq 4000 RNA sequencing. Sequence quality was verified by FastQC (http://www.bioinformatics.babraham.ac.uk/projects/fastqc/) after the deletion of inappropriate reads. *De novo* assembly of transcriptome was performed using Trinity 2.4.0 (Grabherr et al., 2011). The transcripts were grouped according to the output of Trinity, and the longest transcript sequences in each group were selected as ‘gene’ sequence (unigene). Six public databases, including the National Centre for Biotechnology Information nonredundant, GO, Kyoto Encyclopaedia of Genes and Genomes, Protein Family, SwissProt, and eggNOG databases, were used for functional annotations *via* DIAMOND (Buchfink et al., 2015) with a threshold E-value of <10^−5^. The depositing data numbers for the transcriptome were GSE148484 in NCBI database.

### RNA Sequencing (RNA-Seq) Data Analysis

The expression level for unigenes were normalised by transcripts per kilobase of exon model per million mapped reads (TPM) to identify the differentially expressed genes (DEGs) between MT-treated and untreated wax apple samples (Mortazavi et al., 2008). The DEGs were selected with |log_2_(fold change)| ≥ 1 and with statistical significance (*P* < 0.05) by R package edgeR (Robinson et al., 2010). False discovery rate (FDR) was used to determine the threshold of *P* value in multiple testing (Benjamini and Yekutieli, 2001). Three biological replicates were performed to determine the reliability of the DEGs. A volcano plot was drawn with GraphPad Prism 8.0.1 to show the overall DEG distribution. OmicShare tools (http://www.omicshare.com/tools) were used to classify all significantly DEGs into GO functions (i.e., biological process, molecular function, and cellular component). Gene expression profiles determined *via* cluster analysis were drawn using eGPS 1.6.1 software (Yu et al., 2019).

### Quantitative PCR Validation and Expression Analysis

Real-time quantitative PCR (RT-qPCR) was used to confirm the gene expressions related to cell membrane decomposition and modification (*SsPG1*, *Sscel61a*, and *Sscel61b*), antioxidant enzyme (*SsSod*, *SsCAT1*, and *SsSOD2*) and other antioxidant pathways (*SsTrx-2* and *SsTXN2*). RNA was extracted using an RNA preparation pure plant kit (Tiangen, Beijing, China) according to the manufacturer’s instructions. Then, 1 mg of total RNA was reverse transcribed into cDNA using PrimeScript RT reagent kit (TaKaRa, Dalian, China). RT-qPCR was performed using TB Green™ Premix Ex Taq™ II (TaKaRa, Dalian, China). The specific quantitative PCR primers listed in **Supplementary Table S1** were designed using the online software Primer 3 (http://bioinfo.ut.ee/primer3-0.4.0/). The *SsSKIP16* gene, which is highly expressed and unchanged, was used as the internal reference gene. The experiment was repeated three times, and the results were calculated *via* the 2^−ΔΔCt^ method (Livak and Schmittgen, 2001). All experiments were performed with three independent biological replicates.

### Statistical Analysis

This experiment was based on a complete random design and three biological replicates. All values were expressed as mean ± standard deviation (SD). ANOVA was performed using SPSS software. Asterisks show that the values were significantly different between control and 800 µM MT treatment at the same storage time (* *P* < 0.05, ** *P* < 0.01, *** *P* < 0.001).

## Results

### Morphological Changes, Decay Incidence, and Weight Loss Rate

**Figure 1:**
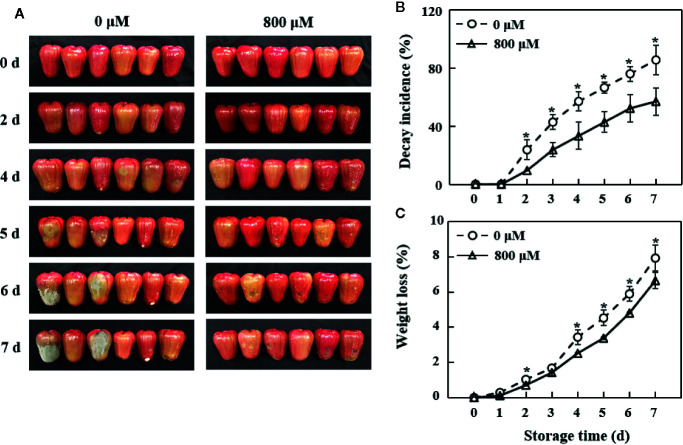
Changes in morphology **(A)**, decay incidence **(B)**, and weight loss rate **(C)** in wax apples treated with MT or diH_2_O (control) during storage. Each data point represents the mean (with SD bar) of three replicates. Asterisks show that the values between control and MT treatment are significantly different (*P < 0.05) at the same storage time.

The fruit in the control group showed morphological discoloration and decay on pericarp with a decay incidence of 57.1% on the 4th day, whereas the decay incidence was 33.3% in the MT treatment group. The visible difference between the control and exogenous MT treatment groups became more obvious on the 5th day (**Figure 1A**). The decay incidence of pericarp gradually increased with storage time (**Figure 1B**). The weight loss rate in the MT treatment group was significantly lower (*P* < 0.05) than that in the control group (**Figure 1C**).

### Reactive Oxygen Species Production, Malondialdehyde Content, and Liquid Oxygen Activity

**Figure 2:**
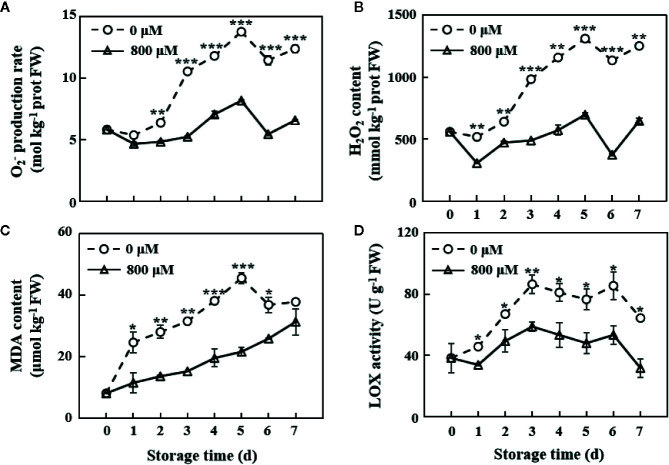
Changes in O_2_^−^ production rate **(A)**, H_2_O_2_ content **(B)**, MDA content **(C)**, and LOX activity **(D)** in the pulp tissues of wax apples treated with MT or diH_2_O (control) during storage. Each data point represents the mean (with SD bar) of three replicates. Asterisks show that the values between the control and MT treatment groups are significant differences (*P < 0.05, **P < 0.01, ***P < 0.001) at the same storage time.

The ROS index, including O_2_^−^ production and H_2_O_2_ content, increased with the increase in storage time. In this study, MT treatment remarkably inhibited the accumulation of these ROSs (**Figures 2A, B**). On the 7th day, O_2_^−^ production rate and H_2_O_2_ content in MT-treated fruit decreased by 46.8 and 48.2% compared with those in the control group, respectively (**Figures 2A, B**). MDA content was substantially decreased by MT treatment (**Figure 2C**). The largest difference in O_2_^−^ production rate and H_2_O_2_ and MDA contents between the MT treatment and control groups was observed on the 5th day of storage. LOX activity in the control group increased in the first 3 days, declined on the 4th and 5th days, slightly increased on the 6th day, and rapidly declined on the 7th day. LOX activity in the MT treatment group displayed the same trend as that in the control group, except that the activity decreased on the 1st day of storage. However, MT treatment significantly (*P* < 0.05) inhibited LOX activity during the whole storage period (**Figure 2D**).

### Activity of Antioxidant Enzymes

**Figure 3:**
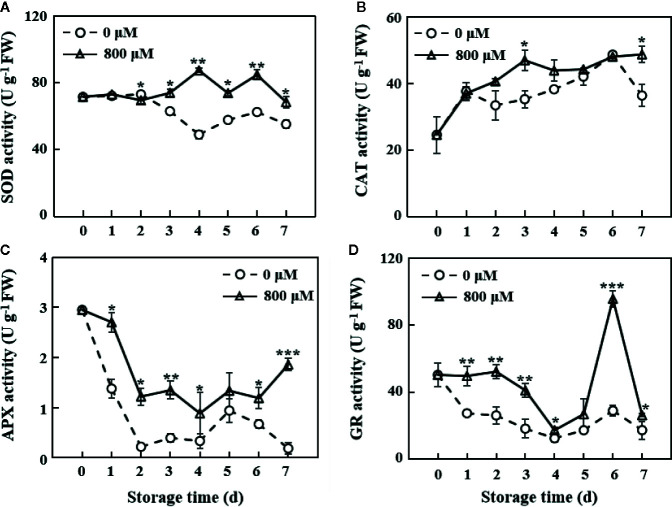
Changes in SOD **(A)**, CAT **(B)**, APX **(C)**, and GR **(D)** activities in the pulp tissues of wax apples treated with MT or diH_2_O (control) during storage. Each data point represents the mean (with SD bar) of three replicates. Asterisks show that the values between the control and MT treatment groups are significant differences (*P < 0.05, **P < 0.01, ***P < 0.001) at the same storage time.

The MT treatment group showed similar SOD activity with the control group on the first 2 days. A fluctuating change in SOD activity was observed on the 3rd day. However, MT-treated fruit showed a significantly higher SOD activity than the controls from the 2nd day of storage (*P* < 0.05 on the 2nd, 3rd, 5th, and 7th days; *P* < 0.01 on the 4th and 6th days; **Figure 3A**). CAT activity showed a continuous upward trend in the MT treatment group during the 7 days of storage but decreased after the 1st and 6th days of storage in the control group. CAT activity was significantly higher (*P* < 0.05) in the MT treatment group than that in the control group on the 3rd and 7th days (**Figure 3B**). APX activity in the control and MT-treated fruit rapidly decreased in the first 2 days of storage. Fruit in the control group showed activity changes opposite to those of MT-treated fruit on the 7th day (**Figure 3C**). Overall, APX activity in the MT treatment group was significantly higher than that in the control group (*P* < 0.05). GR activity in the MT treatment group was significantly higher than that in the control group (*P* < 0.05). On the 6th day, GR activity in the MT treatment group reached the maximum value (95.8 U g^−1^), which was 3.3-fold higher than that in the control group (**Figure 3D**). Collectively, MT-treated fruit showed higher activity of antioxidant enzymes (SOD, CAT, APX, and GR) throughout the storage period compared with the controls (**Figure 3**).

### Jasmonic Acid and Salicylic Acid Contents

**Figure 4:**
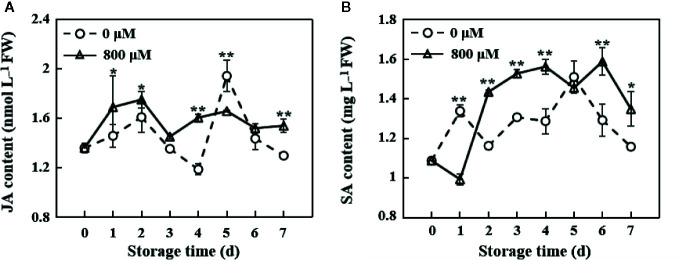
Changes in JA **(A)** and SA **(B)** contents in the pulp tissues of wax apples treated with MT or diH_2_O (control) during storage. Each data point represents the mean (with SD bar) of three replicates. Asterisks show that the values between the control and MT treatment groups are significant differences (*P < 0.05, ** *P* < 0.01) at the same storage time.

JA and SA play important roles in the pathogen resistance. JA content increased in the early storage period in MT-treated fruit, reached the maximum value on the 2nd day, decreased, and maintained a relatively stable level. JA content in the control group was lower than that in the MT treatment group, except on the 5th day (**Figure 4A**). SA content in the MT treatment group was significantly lower than that in the control group on the 1st day (*P* < 0.01). However, SA content in the MT treatment group was significantly higher than that in the control group from the 2nd day to the 7th day of storage, except on the 5th day (*P* < 0.01 on the 2nd, 3rd, 4th, and 6th days; *P* < 0.05 on the 7th day; **Figure 4B**).

### Transcriptome Sequencing of Wax Apple

**Figure 5:**
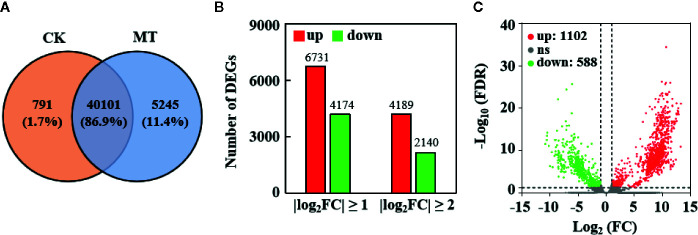
Transcriptome sequencing results in MT-treated and untreated wax apples. **(A)** Distribution of unigene numbers in the control group (left) and MT-treated group (right). **(B)** Statistics on the number of DEGs. X-axis represents the different screened thresholds, and Y-axis is the number of DEGs. Red bar denotes the up-regulated genes, and green bar represents the down-regulated genes. **(C)** Significantly DEGs in the control and MT treatment groups. Green points represent significantly down-regulated DEGs, red points indicate significantly up-regulated DEGs, and gray points mean insignificantly DEGs. The thresholds used are FDR ≤ 0.05 and |log_2_(FC)| ≥ 1.

RNA-seq was performed *via* high-quality transcriptome sequencing of six libraries to investigate the expression profile of genes in MT-treated and untreated wax apples (**Supplementary Table S2**). After filtering out the low-quality reads, 53,860,011 and 54,379,435 clean reads were obtained from the control and MT treatment groups, respectively. A total of 100,980 transcripts and 46,137 nonredundant genes (unigenes) were obtained through Trinity *de novo* assembly (**Supplementary Table S3**). A total of 40,101 genes (86.9% of the total genes) were expressed in the control and MT treatment groups, whereas 791 (1.7%) and 5245 (11.4%) genes were specially expressed in the control and MT treatment groups, respectively (**Figure 5A**).

### Differential Expression Analysis of Wax Apple Genes

**Figure 6:**
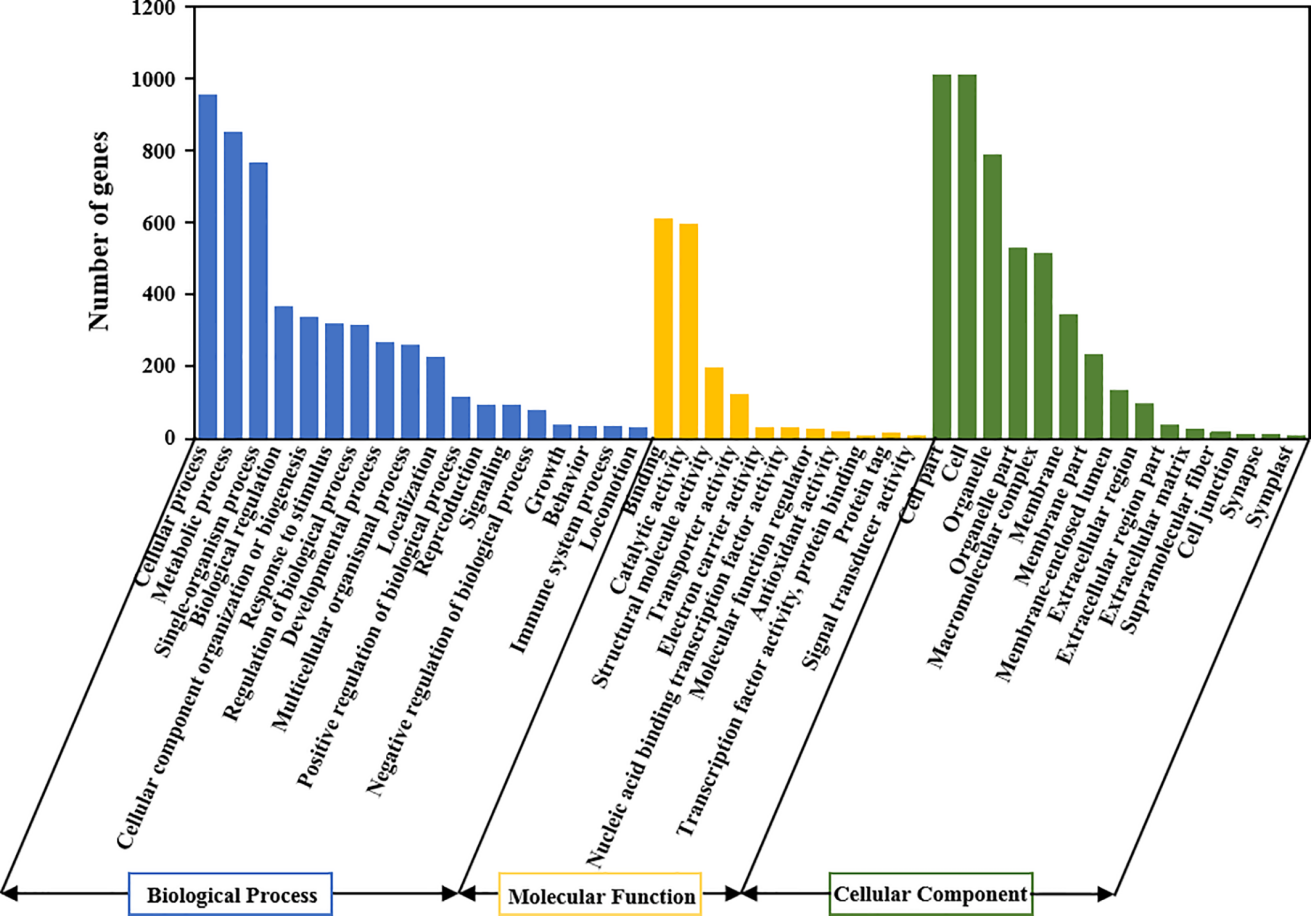
GO classification of all DEGs in the control and treatment groups of wax apples. X-axis is the GO terms, and Y-axis represents the number of significantly DEGs. All GO terms are summarised in three main categories: biological processes, molecular functions and cellular components.

We compared the expression profile between the MT-treated and control fruit to explore the effect of MT treatment on gene expression in wax apple. The numbers of up- and down-regulated genes are shown in **Figure 5B**. A total of 10,905 and 6,329 genes were considered DEGs at |log_2_(FC)| ≥ 1 and ≥ 2, respectively (**Figure 5B**). The volcano map displays the distribution of significantly DEGs with a threshold of |log_2_(FC)| ≥ 1 and FDR < 0.05, and the numbers of up- and down-regulated genes were 1,102 and 588, respectively (**Figure 5C**). We performed GO enrichment analysis for these 1,690 significantly DEGs to obtain their functional distribution (**Figure 6** and **Supplementary Table S4**). These significantly DEGs were classified into three categories: biological processes, molecular functions, and cellular components. For biological processes, most genes were divided into ‘cellular process’ and ‘metabolic process’ subcategories. For molecular functions, 611 and 596 genes were assigned to ‘binding’ (GO:0005488) and ‘catalytic activity’ (GO:0003824), respectively. For cellular components, 21.1% of the genes were classified as ‘cell part’ (GO:0044464), which was the same as ‘cell’ (GO:0005623). GO classifications related to post-harvest preservation were screened. Results indicated that many of these genes were enriched in oxidoreductase activity (GO:0016491) and play a role in oxidation-reduction (GO:0055114) (**Table 1**). Additionally, these gene expressions are displayed in the heatmap (**Supplementary Figure S1**) and listed in **Supplementary Table S5**.

**Table 1:** GO enrichment for differentially expressed genes (DEGs) related to post-harvest wax apple.

GO ID	Description	Type	Gene number	P value	FDR
GO:0003954	NADH dehydrogenase activity	MF	34	***	***
GO:0004601	Peroxidase activity	MF	16	**	*
GO:0006116	NADH oxidation	BP	5	**	*
GO:0016209	Antioxidant activity	MF	20	**	*
GO:0016491	Oxidoreductase activity	MF	211	***	***
GO:0016684	Oxidoreductase activity, acting on peroxide as acceptor	MF	16	**	*
GO:0030964	NADH dehydrogenase complex	CC	39	***	***
GO:0042744	Hydrogen peroxide catabolic process	BP	9	**	*
GO:0045454	Cell redox homeostasis	BP	16	*	*
GO:0055093	Response to hyperoxia	BP	4	**	*
GO:0055114	Oxidation-reduction process	BP	184	***	***
GO:0070482	Response to oxygen levels	BP	12	**	*
GO:0072593	Reactive oxygen species metabolic process	BP	18	*	*
GO:1990204	Oxidoreductase complex	CC	70	***	***

BP, biological process; MF, molecular function; CC, cellular component; FDR, false discovery rate.

*P/FDR < 0.05, **P/FDR < 0.01, ***P/FDR < 0.001

### Quantitative PCR Validation for RNA-Seq Data

**Figure 7:**
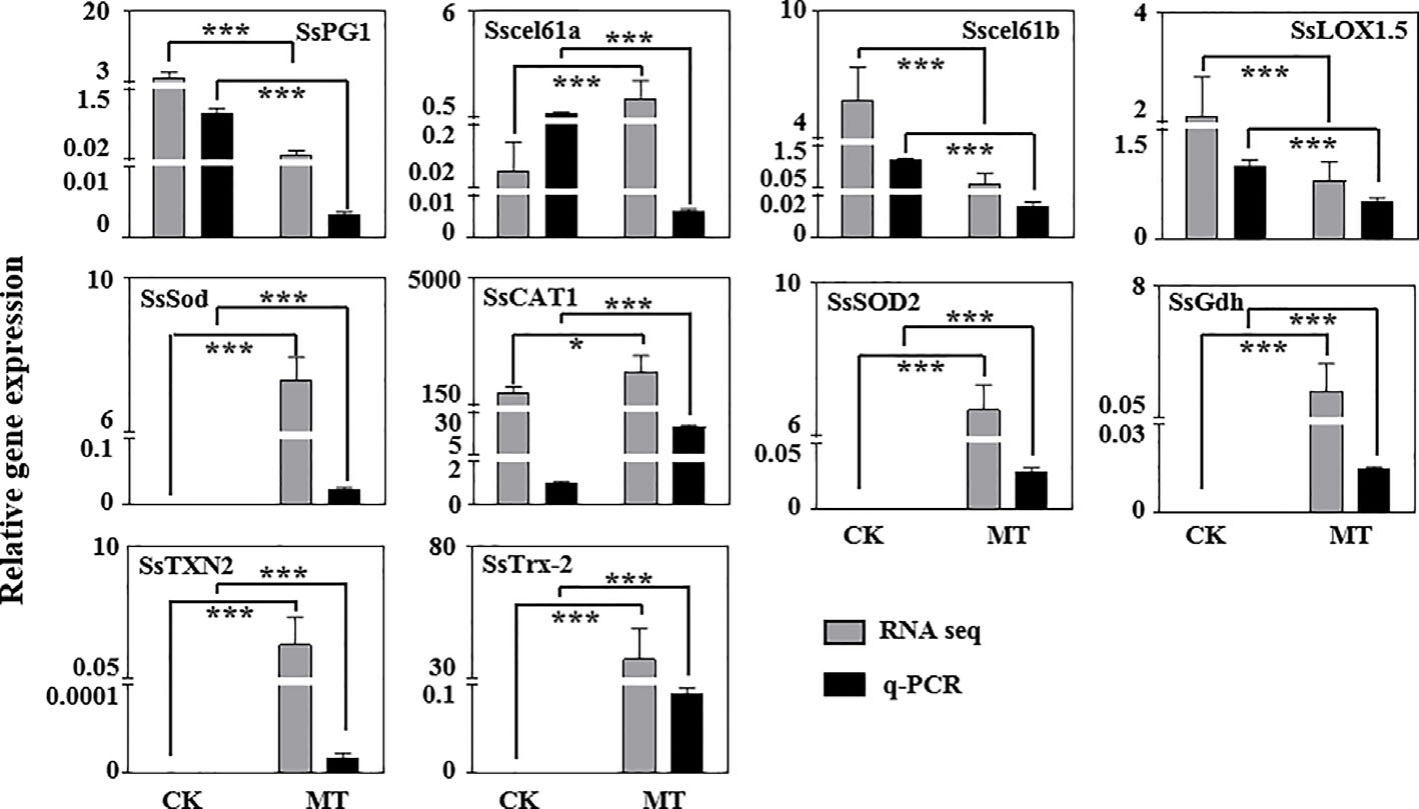
Validation of RNA-seq data *via* RT-qPCR. These genes are associated with cell wall modification, active oxygen metabolism and ROS scavenging system. The *SsSKIP16* gene, which is highly expressed and unchanged, was used for standardisation. CK and MT indicate the control and MT treatment groups on the 5th day, respectively. Gray and black bars represent RNA-seq data and q-PCR experimental results, respectively. Each data point represents the mean (with SD bar) of three replicates. Asterisks show that the values between the CK (control) and MT treatment groups are significant differences (*P < 0.05, ***P < 0.001).

We investigated the expression of several significantly DEGs related to post-harvest preservation *via* RT-qPCR to confirm the validity of the RNA-seq data. Most of the RNA-seq data were consistent with the RT-qPCR results (**Figure 7**). However, the expression of *Sscel61a* gene between RNA-seq and RT-qPCR were opposite probably due to the false positive of the transcriptome itself. Cell wall decomposition and modification-related genes (*SsPG1*, *Sscel61a*, and *Sscel61b*) and lipid peroxidation-related gene (*SsLOX*) were down-regulated by exogenous MT treatment in wax apple. Antioxidant-related genes (*SsSod*, *SsCAT1*, and *SsSOD2*), AsA biosynthesis gene (*SsGdh*), and other antioxidant pathway genes (*SsTrx-2* and *SsTXN2*) were more expressed in the MT treatment group than in the control group.

## Discussion

As a rich source of fructose and vitamin C, wax apple fruit has great potential benefits to human health (Chen et al., 2017). However, wax apples experience water loss and become prone to diseases after picking. Consequently, their appearance becomes unpalatable, which affects its shelf life and market value. In this study, soaking with MT effectively reduced the rate of water loss, alleviated the decay, maintained the appearance, and prolonged the shelf life of wax apples (**Figure 1**). The optimal MT concentration for post-harvest preservation of wax apple (800 µM) is quite higher than that for tomato (50 μM; Sun et al., 2015; Sun et al., 2016), peaches (100 μM; Cao et al., 2016; Gao et al., 2016; Cao et al., 2018; Gao et al., 2018), and pear (Zhai et al., 2018). By contrast, 500, 1,000, and 10,000 µM are the best MT concentration for cassava (Ma et al., 2016), strawberry (Liu et al., 2018), and potato (Zhang et al., 2017), respectively. These results indicated that the amounts of optimal concentration for MT treatment are quite different, which may be related to the difference of species and MT treatment methods.

A dynamic equilibrium relationship exists between ROS production and removal (including O_2_^−^, ^−^OH and H_2_O_2_) under normal growth conditions, but the relationship is broken after the fruits are picked because of rapid ROS accumulation (Sun et al., 2018). In the present study, MT treatment remarkably reduced O_2_^−^ production rate and H_2_O_2_ content in post-harvest wax apples (**Figures 2A, B**). Under the catalysis of LOX enzyme, ROS causes the oxidation of unsaturated fatty acids in the lipid membrane to form conjugated peroxy fatty acids, which may further destroy the structure of the cell membrane and eventually lead to membrane peroxidation and degradation (Chomkitichai et al., 2014). MDA, the final product of membrane lipid peroxidation, is commonly used as an effective indicator of the extent of membrane lipid peroxidation (Dhindsa et al., 1981). The gene expression of *SsLOX1.5* in the MT treatment group was down-regulated (**Figure 7**), and LOX enzyme activity and MDA content were reduced by MT treatment (**Figures 2C, D**). Thus, MT could effectively reduce membrane lipid peroxidation and improve the antioxidant capacity of wax apples. Additionally, the expression levels of cell wall-degrading enzyme genes *SsPG1*, *Sscel61a*, and *Sscel61b* were reduced by MT treatment in wax apple (**Figure 7**), which was consistent with the finding in the pear (Zhai et al., 2018). These results suggested that MT treatment may possibly maintain the firmness of post-harvest wax apples and delay fruit softening.

The oxidative damage of several important molecules leads to the destruction of normal metabolism of cells, but plants also contain antioxidant systems to remove excess ROS (Reiter et al., 1998). Exogenous MT treatment could remarkably increase the antioxidant enzyme activities of SOD, CAT, APX, and GR (**Figure 3**). Molecular level analysis showed that the antioxidant enzyme genes (*SsSod*, *SsCAT1*, and *SsSOD2*) were not expressed in the control group but highly expressed in the MT treatment group (**Figure 7**), suggesting that MT can reduce oxidative damage, maintain complete cell membrane structure, and function and delay post-harvest decay of wax apples. RNA-seq analysis showed that numerous genes participated in the antioxidant term in post-harvest wax apples, especially the ‘antioxidant activity’ (GO:0016209) and ‘ROS metabolic process’ (GO:0072593) (**Figure 6** and **Table 1**). Some DEGs exhibited obvious clustering in ROS metabolism and antioxidant enzyme synthesis, indicating that MT plays an important role in promoting post-harvest preservation by increasing the antioxidant activity in wax apples.

As an important gene in the ascorbate-glutathione (AsA-GSH) cycle, *SsGdh* was expressed in MT-treated wax apples but not in the control group on the 5th day after MT treatment (**Figure 7**), indicating that AsA-GSH may still function as an important antioxidant system in MT-treated wax apples but not in the control group. The gamma aminobutyric acid (GABA) pathway can extend thetricarboxylic acid cycle, provide sufficient ATP and carbon skeleton for cells, relieve oxidative damage, and delay the ageing of post-harvest fruits and vegetables (Aghdam et al., 2019; Sharafi et al., 2019). The present results showed that GABA shunt pathway genes *SsTXN2* and *SsTrx-2* were expressed in post-harvest MT-treated wax apples but not in the control fruit (**Figure 7**), suggesting that the GABA pathway may provide ATP and clear excess H_2_O_2_ in MT-treated wax apples.

JA and SA act as important signaling molecules and play roles in plant growth and development and stress responses (Creelman and Mullet, 1995; Shi and Zhu, 2008). Recent studies proved that MeJA treatment could prolong the post-harvest life of cassava, reduce post-harvest physiological deterioration (PPD), and regulate the storage quality (Liu et al., 2019). Also, exogenous SA could improve the color, keep the content of chlorophyll, ascorbic acid, and maintain the good quality of post-harvest asparagus (Wei et al., 2011). In our study, we found that JA content in the MT treatment wax apple was significantly higher than that in the control group on the 1st, 2nd, 4th, and 7th day during storage, except on the 5th day (**Figure 4A**). And similarly, SA content in the MT treatment group was also significantly higher than that in the control group from the 2nd day to the 7th day, except on the 5th day (**Figure 4B**). Arnao and Hernández-Ruiz reported that MT mainly acts on the upstream of defense genes to promote the synthesis of JA and SA (Arnao and Hernández-Ruiz, 2018). These results suggested that MT treatment may improve the disease resistance of post-harvest wax apple by increasing the accumulation of the content of JA and SA.

**Figure 8:**
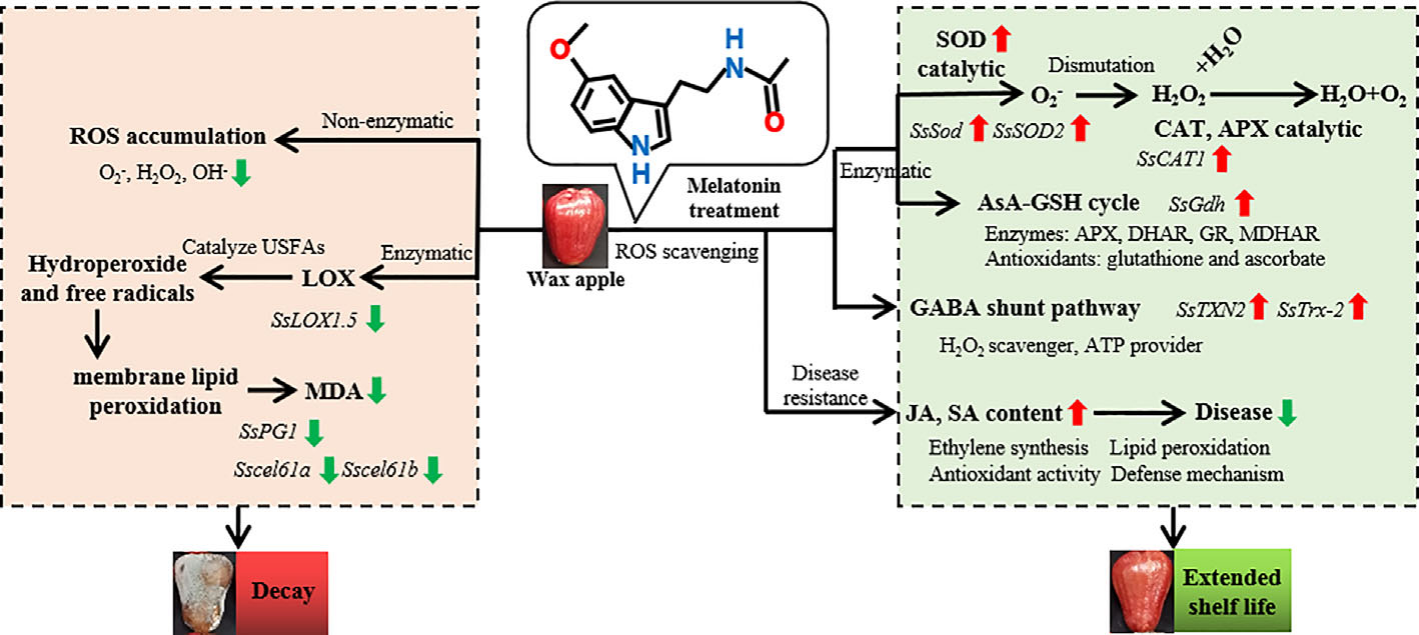
Mechanism model of exogenous MT in delaying the post-harvest decay of wax apples. The left part displays the ROS accumulation pathway. The synthesis of ROS can be divided into enzymatic and nonenzymatic pathways. Nonenzymatic pathways can directly lead to ROS accumulation. In enzymatic pathways, LOX enzyme can catalyse unsaturated fatty acids to form hydroperoxides and free radicals, eventually leading to membrane lipid peroxidation. All of the reactions will cause the decay of post-harvest wax apples. The right part indicates the MT action pathway. The main function of MT is to remove excess ROS though antioxidant enzymes and antioxidant pathway. The antioxidant enzyme pathway includes SOD, CAT, APX, and AsA-GSH cycles. ROS can be also removed through the GABA shunt pathway. The increase in JA and SA contents will also decrease the risk to post-harvest diseases. Red and green arrows indicate the up-regulation and down-regulation of expression levels under exogenous MT treatment, respectively. Italics represent gene expression during post-harvest preservation process.

In summary, MT eliminated excessive ROS and delayed the post-harvest decay of wax apples. The model in **Figure 8** briefly describes ROS accumulation and the potential mechanism of MT in post-harvest wax apples. The model shows that the post-harvest decay of wax apples is closely related to ROS accumulation and cell membrane damage. MT removes excess ROS in wax apples through the collaborative functions of antioxidant enzymes, such as SOD, CAT, APX, and GR, and the GABA shunt pathway. In addition, the increase in JA and SA contents reduced the visible incidence of post-harvest diseases in wax apples.

## Data Availability Statement

The original contributions presented in the study are publicly available. This data can be found here: NCBI/GSE148484. (https://www.ncbi.nlm.nih.gov/geo/query/acc.cgi?acc=GSE148484.

## Author Contributions

TX and ZL conceived and designed the experiments. YC, CZ, YL, TD, MZ, XD, and LZ performed the experiments. YC and YZ performed the statistical analysis of the data. TX and YC wrote the manuscript. YZ and GN helped to revise the manuscript. All authors contributed to the article and approved the submitted version.

## Funding

This work was supported jointly by the Natural Science Foundation of Jiangsu Higher Education Institutions of China (19KJA510010), the Priority Academic Program Development of Jiangsu Higher Education Institutions (PAPD) and Postgraduate Research & Practice Innovation Program of Jiangsu Province (SJKY19_2047).

## Conflict of Interest

The authors declare that the research was conducted in the absence of any commercial or financial relationships that could be construed as a potential conflict of interest.
